# Myocardial Edema Revisited in a New Paradigm of Cardiac Electrical Microcurrent Application in Heart Failure

**DOI:** 10.1089/bioe.2021.0021

**Published:** 2021-09-09

**Authors:** Jesus Eduardo Rame, Johannes Müller

**Affiliations:** ^1^ Department of Medicine, Jefferson Heart Institute, Philadelphia, Pennsylvania, USA.; ^2^ Department of Bioelectricity and Medical Research, Berlin Heals, Berlin, Germany.

**Keywords:** cardiac microcurrent, myocardial edema reduction, electro-osmosis

## Abstract

Undisturbed bioelectricity is a prerequisite for normal organ function. This is especially true for organs with high electrical activity such as the heart and the nervous system. Under clinical conditions, however, this can hardly be determined in patients with disturbed organ function and is therefore largely ignored. Here, based on clinical data, we will discuss whether the direct application of an external electric current (in the physiological μA range) together with an electrical field to hearts with impaired pump function can explain the functional improvement of the hearts by edema reduction triggered by electro-osmosis.

The application of an electrical nonpulsating microcurrent to the heart of patients with moderate ambulatory New York Heart Association (NYHA) Class III heart failure can result in a rapid improvement in cardiac performance. A recently reported longitudinal study identified an increased left ventricular ejection fraction with a reduction in end-systolic and end-diastolic left ventricular size along with an increase in 6-min walk, NYHA functional class, and quality of life.^1^

There is a rapid functional improvement that can be demonstrated after only a few hours of microcurrent application (Fig. 1). If the early effects on the myocardium from the application of a microcurrent are on this time scale (hours), we know that the attenuation of inflammation cannot explain the acute response being observed.^2,3^ This short report proposes a mechanism of action for the early recovery of cardiac function that has been observed in the initial experience of microcurrent application.

**FIG. 1.**
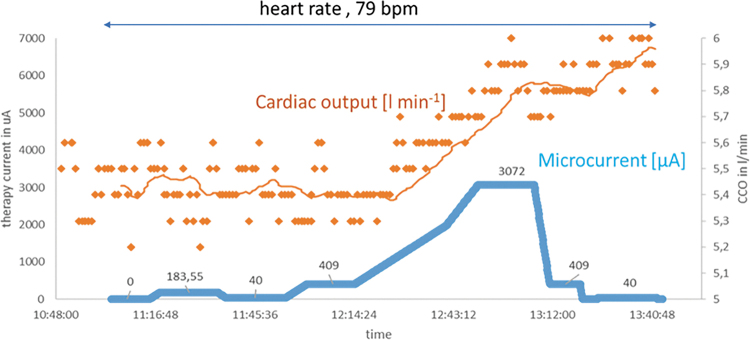
Proportionality of electrical current application and cardiac output. In a sheep model with surgically implanted electrodes and measuring cardiac output through a CCO monitoring system (Vigilance; Baxter Healthcare Corporation's Edwards Critical-Care Division), we demonstrate the time correlation of microcurrent application and cardiac output. In this model, an increase in microcurrent is paralleled by an increase in cardiac output. Given the immediate stroke volume response, a mechanism that could explain the impact of microcurrent on the myocardium is a reduction in myocardial fluid content. Consistent with this observation is that a reduction in microcurrent does not lead to an immediate drop in CCO because the process of fluid retention is subject to a physiological delay of reaccumulation of fluid into the myocardium. *Blue line*, electrical microcurrent; *ochre yellow* line, result of a moving average smoothing process of the individual measuring points with a *n* = 25. CCO, continuous cardiac output.

Although it receives little attention because of the lack of direct therapeutic interventional options and because its exact effect on the impairment of cardiac function is unknown, myocardial edema plays an essential role in the physiology of cardiac function and the pathology of heart failure.^4–6^ Physical forces govern fluid filtration to a certain degree. Myocardial edema, the excess accumulation of fluid in the myocardial interstitium, develops when there is an imbalance between filtration from the coronary microvasculature, removal of interstitial fluid through lymphatic vessels, and epicardial transudation.^7,8^ Under pathophysiological conditions, large quantities of proteins accumulate in the interstitial space, producing an osmotic/oncotic sucking force that drives fluid flow into the tissue and prevents fluid absorption back into the circulation. This chain of events creates the essential conditions for the formation of edema.^9^

Alteration of capillary permeability by inflammation (capillary leakage), loss of charge of the highly negatively charged glycocalyx, the inability of lymphatic system to drain the increased fluid, or a combination of these factors leads to the formation of myocardial edema.^10–14^ Myocardial edema impairs myocyte contractility and is responsible for systolic and diastolic dysfunction.^15–17^ Experimental data suggest that when myocardial fluid content is increased by only a few percent, cardiac output can easily be compromised by >15%.^15,18,19^ Recently, a murine model of increased coronary sinus pressure inducing myocardial edema demonstrated significant cardiac contractile dysfunction within 24 h and the subsequent development of epicardial inflammation and fibrosis in the left ventricle.^20^ Postmortem data of patients who died from cardiovascular diseases show a 30% increase in heart weight for men and a 22% increase for women, largely because of myocardial edema.^21,22^

Patients have been known to experience massive myocardial edema during acute myocarditis, requiring immediate intervention with mechanical assist pumps.^23–26^ Moreover, beyond acute inflammatory cardiomyopathies associated with severe myocardial edema, a number of other cardiac conditions predispose to interstitial edema in the myocardium. After myocardial infarction, myocardial edema remains detectable for as long as 6 months.^27^ Untreated acute myocardial edema is at least partially responsible for early death after myocardial infarction.^28,29^ Consequently, cardiac contractile (systolic) dysfunction is probably an important factor contributing to the insufficient cardiac lymphatic drainage observed after myocardial infarction.^30^ Myocardial edema has been detected among patients with arrhythmias who require support with defibrillators.^31^ Patients with diabetes are more likely to experience long-lasting myocardial edema after infarction than are patients without diabetes.^32^ Pulmonary hypertension has been associated with myocardial edema in patients with impaired left ventricular function.^33^ Across the spectrum of heart failure, patients with dilated cardiomyopathy and reduced ejection fraction (HFrEF), mid-range ejection fraction (HFmEF), as well as patients with preserved ejection fraction (HFpEF), can exhibit substantial degrees of interstitial edema in the heart.^8,34–37^

Furthermore, because of capillary leakage and overstrained lymphatic drainage, any type of myocardial or systemic inflammation, including SIRS and sepsis, can probably also be associated with various degrees of myocardial edema.^38^ For adequate drainage of the interstitium and transport of lymph, the lymphatic vessels require normal pumping function of the heart within certain limits. Thus, impaired cardiac function contributes to impaired lymphatic flow and leads to myocardial edema^16,39^

The fatal feature of myocardial edema formation is that it not only impedes the pumping function of the heart in a purely mechanical manner but also exerts a proinflammatory effect, induces fibrosis, stimulates exuberant collagen synthesis in the interstitium by upregulating transforming growth factor-beta and the pro-collagen type I and type III, resulting in increased collagen deposition.^40,41^ In addition, there is evidence indicating that the excitation-contraction uncoupling induced by myocardial edema causes contractile dysfunction.^17,42^ Experimental removal of edema leads to an immediate improvement in the contractility of the heart.^30^

Thus, a therapeutic strategy is needed to allow patients to escape the vicious circle of edema, inflammation, and fibrosis associated with the frequently observed decline in cardiac function. This strategy should improve lymphatic flow independent of capillary permeability (negatively charged glycocalyx) and pressure conditions (hydrostatic and colloid osmotic) in the capillaries and the interstitium.^43^

Electrorheology, electrokinetics, and, in particular, electrophoresis and electro-osmosis describe the transport mechanisms of fluids or charged particles, mechanisms that are induced by the application of an electrical field or a direct current, in which an electrical field is inherent. Electrophoresis is well known in medicine as a method of inducing the migration of charged colloidal particles or molecules suspended in a solution by means of an electrical field, with the intention of separating and analyzing them in turn. In addition, within a living organism electrophoresis plays a relevant role in the distribution of molecules.^44,45^

Although electro-osmosis is well known in biophysics and biology, it is hardly known in medicine. It describes the phenomenon of the movement of a liquid through a capillary vessel bearing a surface charge caused by an electrical field parallel to the surfaces of the vessel.^34,46,47^ Electro-osmotic flow may occur through small-channel structures such as gap junctions, capillaries, cells, and cell membranes across a heterogenous group of tissues. In particular, electro-osmotic flow can be found in areas of high electrical activity.^48–50^ In addition to the flow generated by pulsatile or osmotic and hydrostatic pressure differences, electrokinetic flow is a relevant transport mechanism in even the smallest lymphatic and blood vessels.^51^ Thus, edema formation can be interpreted in part as a disturbed electrical environment generating a potential gradient in the myocardium. This environment includes the negatively charged intraluminal layer (glycocalyx) of the fluid exchange vessels; this layer controls the permeability of the capillary wall and, in turn, the fluid balance and pressure conditions in the interstitium.^5,40,52^

An externally applied weak electric current together with an electrical field can interrupt the vicious circle of edema formation and impaired cardiac performance. It normalizes the electrical environment with the consequence of increasing lymphatic flow by supporting the electrokinetically induced transport of electro-osmosis and electrophoresis and may also influence the charge of the glycocalyx.^50^

The velocity V_EO_ of the electro-osmotic flow in response to an electrical field can be described by the Helmholtz–Smoluchowski relation:

$$V_{EO}=\varepsilon \varepsilon_{0}\zeta V∕\eta ,$$

where ɛ is the permittivity of the medium (blood and lymphatic fluid), ɛ_0_ the dielectric constant of vacuum, ζ is the zeta-potential of the relevant particles in the fluid, V is the strength of the external applied electrical field, and η is the viscosity of the fluid (blood and lymphatic fluid). More details about this process have been previously published.^53^

From this mathematical description, one can roughly estimate the strength of the electrical field that must be applied to achieve a certain velocity of lymphatic drainage. It must be recognized that the applied electrical field is inhomogeneous and cannot always be perfectly aligned in parallel with the vessels. Interestingly, the direct proportionality between flow velocity and external electrical field allows the determination of flow velocity by field strength within certain limits (Fig. 1).

Future translational work using cardiac magnetic resonance imaging to quantitate the degree of myocardial edema will determine if there is a correlation between the cardiac improvement achieved and the degree of resolution of myocardial edema induced by the application of a microcurrent. These studies could have important implications in acute cardiomyopathy syndromes, where the ability to reduce interstitial edema in the myocardium may promote cardiac recovery and prevent patients from progressive heart failure into cardiogenic shock.^25^

Today's heart failure therapy with pulsatile devices does not allow to clearly predict whether a patient will profit from the implantation of such a device. Information on existing edema might be used to stratify patients before implantation of a microcurrent device. Patients without myocardial edema will most likely not show a rapid increase in cardiac function immediately after the implantation; they must wait for the anti-inflammatory effect of long-term application of microcurrent on myocardial function.

Without prejudging clinical confirmation, one could hypothesize that the spectrum of patients with heart failure who benefit from the application of microcurrent directly to the heart may be largely independent of the cause of the disease, depending only on the extent of myocardial edema.

In conclusion, the early improvement in cardiac function after the application of a microcurrent directly to and around the heart could implicate favorable changes in myocardial edema, which heretofore has not been a clinical target in human heart failure.
